# Identification of the distal end of the palisade vessels under sedation: a multicenter prospective study in Japan

**DOI:** 10.1007/s00535-026-02385-6

**Published:** 2026-04-01

**Authors:** Waku Hatta, Tomoyuki Koike, Ayaka Takasu, Satoshi Ono, Fumiaki Ishibashi, Tsutomu Takeda, Chika Kusano, Yuto Shimamura, Masakatsu Fukuzawa, Keiko Yamamoto, Yuji Ino, Naomi Kakushima, Ai Fujimoto, Shiko Kuribayashi, Taro Iwatsubo, Yuji Urabe, Hideki Ishikawa, Tomohiro Nakamura, Naoki Nakaya, Atsushi Masamune, Yoshimasa Miura, Mitsuhiro Fujishiro, Kentaro Sugano

**Affiliations:** 1 Division of Gastroenterology, Tohoku University Graduate School of Medicine, 1-1 Seiryo-Machi, Aoba-Ku, Sendai, Miyagi 980-8574 Japan; 2 Division of Gastroenterology and Hepatology, Department of Medicine, Nihon University School of Medicine, 30-1 Oyaguchi-Kamicho, Itabashi-Ku, Tokyo 173-0034 Japan; 3 Department of Gastroenterology and Gastrointestinal Endoscopy, Tokyo Metropolitan Institute for Geriatrics and Gerontology, 35-2 Sakaecho, Itabashi-Ku, Tokyo 173-0015 Japan; 4 Department of Gastroenterology, International University of Health and Welfare Ichikawa Hospital, 6-1-14 Konodai, Ichikawa, Chiba 272-0827 Japan; 5 Department of Gastroenterology, Juntendo University School of Medicine, 3-1-3 Hongo, Bunkyo-Ku, Tokyo 113-8431 Japan; 6 Department of Gastroenterology, Kitasato University School of Medicine, 1-15-1 Kitasato, Minami-Ku, Sagamihara, Kanagawa 252-0375 Japan; 7 Digestive Diseases Center, Showa Medical University Koto Toyosu Hospital, 5-1-38 Toyosu, Koto-Ku, Tokyo 135-0061 Japan; 8 Department of Gastroenterology and Hepatology, Tokyo Medical University, 6-7-1 Nishi-Shinjuku, Shinjuku-Ku, Tokyo 160-0023 Japan; 9 Department of Gastroenterology and Hepatology, Graduate School of Medicine, Hokkaido University, 15-7 Kita 15-Jo Nishi, Kita-Ku, Sapporo, Hokkaido 060-8648 Japan; 10 Department of Medicine, Division of Gastroenterology, Jichi Medical University, 3311-1 Yakushiji, Shimotsuke , Tochigi 329-0498 Japan; 11 Department of Gastroenterology, Graduate School of Medicine, The University of Tokyo, 7-3-1, Hongo, Bunkyo-Ku, Tokyo 113-8655 Japan; 12 Gastroenterology and Hepatology, Omori Medical Center, Toho University, 6-11-1 Omorinishi, Ota-Ku, Tokyo 143-0015 Japan; 13 Department of Gastroenterology and Hepatology, Gunma University Graduate School of Medicine, 3-39-15 Showamachi, Maebashi, Gunma 371-8511 Japan; 14 Second Department of Internal Medicine, Osaka Medical and Pharmaceutical University, 2-7 Daigakumachi, Takatsuki, Osaka 569-8686 Japan; 15 Department of Gastroenterology, Hiroshima University Hospital, 1-2-3 Kasumi, Minami-Ku, Hiroshima 734-8551 Japan; 16 Molecular-Targeting Prevention, Kyoto Prefectural University of Medicine, 3-1-14 Koraibashi, Chuo-Ku, Osaka 541-0043 Japan; 17 Faculty of Data Science, Kyoto Women’s University, 35 Imakumano Kitahiyoshicho, Higashiyama-Ku, Kyoto 605-8501 Japan; 18 Department of Preventive Medicine and Epidemiology, Tohoku Medical Megabank Organization, Tohoku University, 2-1 Seiryo-Machi, Aoba-Ku, Sendai, Miyagi 980-8575 Japan; 19 Department of Medicine, Jichi Medical University, 3311-1 Yakushiji, Shimotsuke, Tochigi 329-0498 Japan

**Keywords:** Gastroesophageal junction, Distal end of palisade vessel, Proximal end of gastric fold

## Abstract

**Background:**

Although the distal end of palisade vessels (DEPV) has recently been proposed as an anatomically appropriate endoscopic landmark for defining the gastroesophageal junction (GEJ), its evidence under sedation remains limited. This study aimed to evaluate DEPV visualization under sedation, including its detectability and related factors.

**Methods:**

This was a multicenter prospective cohort study conducted at 15 institutions across Japan. Patients underwent real-time endoscopic GEJ observation under sedation in three views: forward view on insertion, retroflex view, and forward view on withdrawal. The primary endpoint was the DEPV identification rate. The secondary endpoints included the DEPV identification rates across three sedation levels and factors associated with unsuccessful DEPV identification.

**Results:**

A total of 638 patients were enrolled. The DEPV identification rate was highest in the forward view on insertion (77.0%). When the retroflex view and/or the forward view on withdrawal were additionally considered, the overall identification rate increased to 85.3%. The identification rate declined with deeper sedation, while combining all three views yielded identification rates ranging from 80.1% under deep sedation to 100% under minimal sedation. In multivariate analysis, risk factors for unsuccessful DEPV identification were deep sedation (odds ratio [OR], 3.83), reflux esophagitis (OR, 2.24–16.37), and incomplete GEJ distention (OR, 8.87), whereas hiatus hernia was protective (OR, 0.52).

**Conclusions:**

This largest multicenter prospective study demonstrated that early DEPV identification during insertion is most effective, whereas deep sedation adversely affects visualization, supporting a standardized approach for routine GEJ assessment.

**Supplementary Information:**

The online version contains supplementary material available at 10.1007/s00535-026-02385-6.

## Introduction

Gastroesophageal junction (GEJ) is an important landmark because it is closely linked to the definition of the distal end of columnar-lined esophagus and gastric cardia [1, 2]. The distal end of palisade vessels (DEPV) and the proximal end of gastric folds (PEGF) are two major endoscopic landmarks for identifying GEJ, but the choice between these two landmarks varies across the guidelines [3–6]. According to a recent international consensus report for GEJ [7], DEPV is more appropriate than the PEGF for defining the GEJ based on the anatomical evidence. This anatomical rationale has been further supported by a recent histological study, which demonstrated that cardiac glands are located within approximately 1 cm proximally and distally from the DEPV as a landmark [8]. Gastric folds can become indistinct in severe gastric atrophy and the PEGF is susceptible to change with air insufflation during endoscopic observation [7]. In contrast, the palisade vessels (PV) are thin, longitudinal vessels located in the mucosal layer of the lower esophageal sphincter, which descends into the submucosa upon entering the cardia [9]; thus, PV are present in a consistent location. However, the DEPV visualization can be impaired by reflux esophagitis or columnar-lined esophagus due to inflammation and appropriate air insufflation is also required for it [10].

Many endoscopic examinations are carried out under sedation in the United States and Europe [11, 12], and the use of sedation is also increasing in Japan [13]. PV become more clearly visible during deep inspiration [14], but it is sometimes difficult to inhale deeply responding to verbal indication, particularly under deep sedation. This limitation may hinder PV visualization and has led to criticism that PV are faint and cannot always be clearly visualized in Western patients [2, 10]. However, reliable data on DEPV visualization under sedation remain scarce. To date, only two single-center studies with small sample sizes (*n* = 82 and 25) have investigated DEPV identification under sedation [14, 15]. Consequently, detailed characteristics of DEPV, including its identification according to sedation level, factors associated with unsuccessful identification, and concordance with PEGF, remain unclear. This study aimed to clarify these aspects by comprehensively evaluating DEPV visualization in a large prospective cohort.

## Methods

### Study design

This was a multicenter prospective cohort study involving 15 institutions across Japan. The study was conducted in accordance with the Declaration of Helsinki and the ethical guidelines for medical and health research involving human subjects in Japan. Prior to patient recruitment, the study protocol was authorized by the Ethics Committee of the Tohoku University Graduate School of Medicine (2023-1-385). The study was registered with the University Hospital Medical Information Network Clinical Trials Registry (UMIN000051868) and was reported in accordance with the Strengthening the Reporting of Observational Studies in Epidemiology (STROBE) statement. Written informed consent was obtained from all participants before enrollment.

### Study participants

Eligibility criteria were: (1) patients scheduled for esophagogastroduodenoscopy (EGD) under sedation in whom the GEJ zone was to be observed, and (2) patients who provided written informed consent after receiving a full explanation of the study. Exclusion criteria were: (1) refusal to participate in the study; (2) a history of surgical resection of the upper gastrointestinal tract; (3) prior endoscopic treatment in the GEJ zone; (4) presence of tumors in the GEJ zone; and (5) patients deemed inappropriate for participation by the investigators.

### Study procedure

Before EGD, baseline information was collected, including height and weight, smoking and drinking history, presence of portal hypertension, and the use of proton pump inhibitors (PPIs) or potassium-competitive acid blockers (P-CABs).

Endoscopic observation for the GEJ under sedation was performed, beginning with a forward view on insertion, followed by a retroflex view and a forward view on withdrawal in variable order. During GEJ observation, examiners instructed patients to take a deep inspiration. In each view, the following parameters were assessed in real time: complete or incomplete GEJ distention, the DEPV score, position of DEPV relative to the squamocolumnar junction, and the position of the PEGF relative to the DEPV. All endoscopic findings were evaluated using white-light imaging without magnification. In addition, data were recorded on the type of endoscope used, sedation level based on the American Society of Anesthesiologists sedation/anesthesia classification [16], use of analgesics and antispasmodics, reflux esophagitis based on Los Angeles (LA) classification with minimal change [17–19], columnar-lined esophagus based on the Prague C & M criteria [10], hiatus hernia based on a previous report [20], and endoscopic gastric atrophy based on the Kimura–Takemoto classification [21]. Sedatives included midazolam, diazepam, haloperidol, and propofol; analgesics included pentazocine and pethidine hydrochloride; and antispasmodics included scopolamine butylbromide, glucagon, timepidium bromide hydrate, and l-menthol.

### Definition of the DEPV score and DEPV identification

**Fig. 1:**
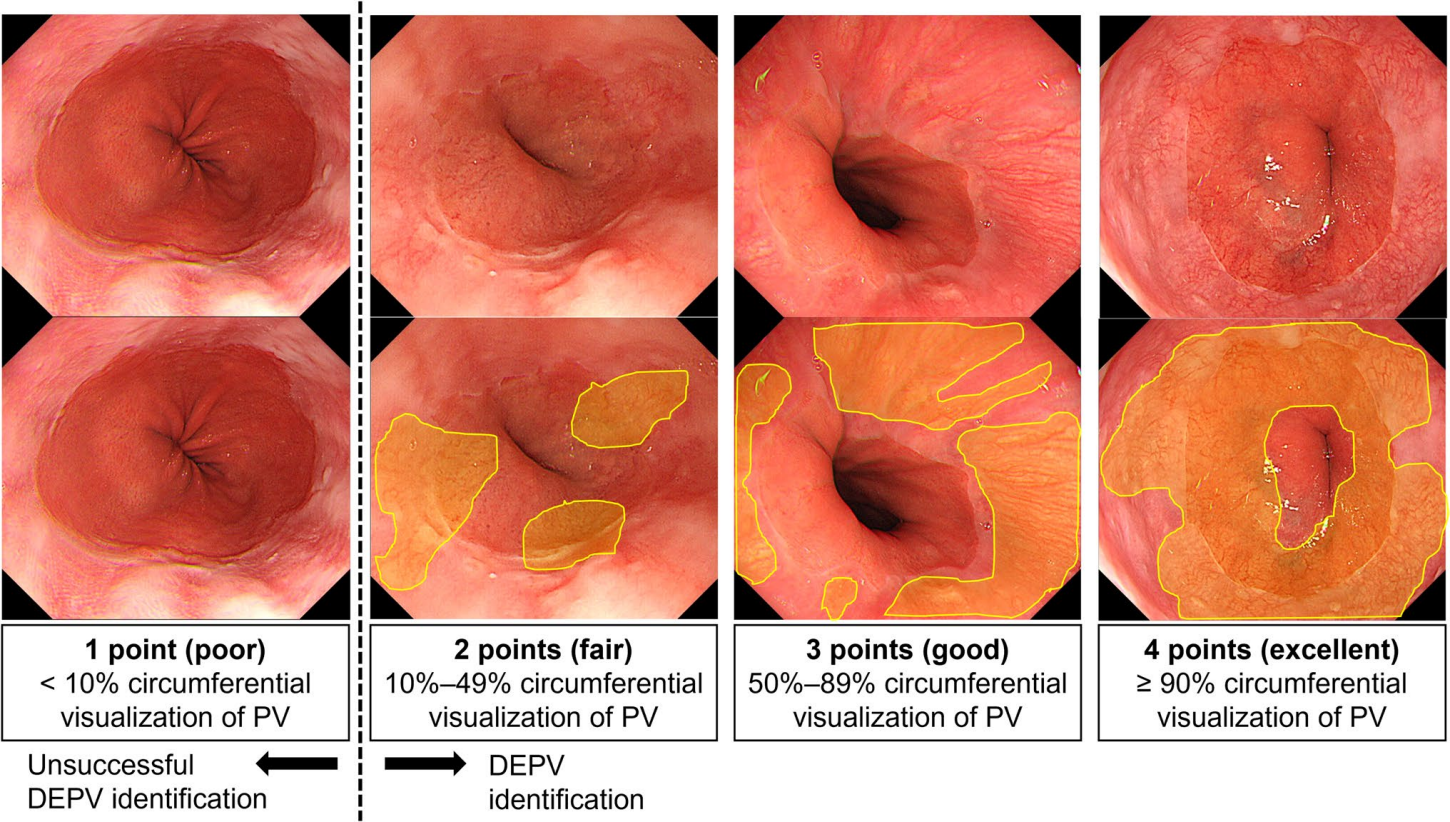
The DEPV score and definition of DEPV identification. DEPV, distal end of palisade vessels; PV, palisade vessels

No standardized methods for assessing the degree of DEPV visualization have previously been reported. Therefore, we established a DEPV scoring system, defined prior to finalization of the study protocol, as follows: 1 point (poor), < 10% circumferential visualization of PV; 2 points (fair), 10%–49% visualization; 3 points (good), 50%–89% visualization; and 4 points (excellent), ≥ 90% visualization (Fig. 1). In this study, DEPV identification was defined as a DEPV score of ≥ 2 because our primary focus was whether the DEPV was visualized at all, rather than the extent of circumferential visualization. A total of 48 endoscopists (44 expert and 4 non-expert endoscopists) were registered as examiners. All examiners had experience with ≥ 3,000 EGD procedures before the start of the study. As a quality control measure, the examiners were trained with example endoscopic images representing each DEPV score before starting this study, and a consensus was reached to evaluate DEPV under appropriate air insufflation.

### Study endpoints

The primary endpoint was the DEPV identification rate assessed by white-light imaging. Secondary endpoints were: (1) DEPV identification rates across three sedation levels (minimal, conscious, and deep sedation), (2) factors associated with unsuccessful DEPV identification, and (3) the concordance rate between PEGF and DEPV, including factors associated with discordance. The primary endpoint was evaluated in three endoscopic views: forward view on insertion, retroflex view, and forward view on withdrawal. Secondary endpoints were evaluated using white-light imaging mainly in the forward view on insertion.

According to the previous studies [7, 10, 14, 22], inadequate air insufflation, reflux esophagitis, and sedation have been associated with unsuccessful PV visualization. Therefore, incomplete GEJ distention, sedation level (minimal vs. conscious and deep), and reflux esophagitis (none vs. minimal change, LA grade A, and LA grade B/C) were considered candidate factors for unsuccessful DEPV identification. In addition, we evaluated age (< 75 vs. ≥ 75 years), sex (female vs. male), body mass index (BMI) (≥ 18.5 to < 25.0 vs. < 18.5 and ≥ 25.0 kg/m^2^), portal hypertension, PPI/P-CAB use, use of analgesics, use of antispasmodics, type of endoscope (standard vs. ultrathin), hiatus hernia, and endoscopic gastric atrophy (none vs. closed type and open type) as potential associated factors. Cut-off values for age and BMI were determined according to official proposals from the Japan Gerontological Society/Japan Geriatrics Society and the World Health Organization classification, respectively [23, 24]. As no previous reports have investigated factors contributing to discordance between PEGF and DEPV, the same candidate variables were assessed for this analysis.

### Sample size calculation

This was an exploratory study, and no reliable prior studies were available for our primary endpoint. To ensure enrollment of an adequate number of patients from all participating institutions, the sample size was set at 500 or more (up to a maximum of 1,000), with a minimum of 25 patients per institution.

### Statistical analysis

Continuous data were expressed as the median and interquartile range. Categorical data were expressed as numbers and percentages, and were compared using the chi-square test. For multiple comparisons (three tests), uncorrected *p* values are presented together with the Bonferroni-adjusted threshold (*p* < α/3 considered statistically significant, where α shows significance level). Logistic regression models were used to evaluate associations of candidate variables with unsuccessful DEPV identification and with discordance between PEGF and DEPV. All variables were included in the multivariate models. Multicollinearity was assessed using variance inflation factors (VIFs), with VIF > 5 indicating multicollinearity [25]. A two-tailed *p* value < 0.05 was considered statistically significant. All analyses were performed using SPSS Statistics for Windows, version 25.0 (IBM Corp., Armonk, NY, USA).

## Results

### Baseline characteristics

A total of 646 patients were initially enrolled between August 2023 and February 2025 (Supplementary Fig. S1). Each institution included at least 25 patients. After the exclusion of 8 patients, 638 were included in the final analysis. Details of the baseline clinical and endoscopic characteristics are shown in Table 1. The median age was 68 years, and minimal, conscious, and deep sedation were administered to 38, 303, and 297 patients, respectively. A total of 621 cases (97.3%) were examined by expert endoscopists certified by the Japan Gastroenterological Endoscopy Society.

**Table 1:** Clinical and endoscopic characteristics of the enrolled patients

	All (*n* = 638)
Age, years, median (IQR)	68 (55–76)
Male sex, *n* (%)	337 (52.8)
Smoking history, *n* (%)	306 (48.0)
Drinking history, *n* (%)	396 (62.1)
BMI, kg/m^2^, median (IQR)	22.5 (20.3–24.6)
Portal hypertension, *n* (%)	15 (2.4)
PPI/P-CAB use, *n* (%)	169 (26.5)
Sedation level, *n* (%)	
Minimal	38 (6.0)
Conscious	303 (47.5)
Deep	297 (46.6)
Analgesic use, *n* (%)	224 (35.1)
Antispasmodic use, *n* (%)	58 (9.1)
Ultrathin endoscope use, *n* (%)	14 (2.2)
Reflux esophagitis, *n* (%)	
None	480 (75.2)
Minimal change	83 (13.0)
LA grade A	57 (8.9)
LA grade B	16 (2.5)
LA grade C	2 (0.3)
Hiatus hernia, *n* (%)	136 (21.3)
Incomplete GEJ distention, *n* (%)	170 (26.6)
Endoscopic gastric atrophy, *n* (%)	
None	319 (50.0)
Closed type	131 (20.5)
Open type	188 (29.5)
Columnar-lined esophagus, *n* (%)	
None	360 (56.4)
Short-segment columnar esophagus	261 (40.9)
Long-segment columnar esophagus	17 (2.7)

IQR, interquartile range; BMI, body mass index; PPI, proton pump inhibitor; P-CAB, potassium-competitive acid blocker; LA, Los Angeles; GEJ, gastroesophageal junction

### DEPV identification rates and the DEPV scores in three endoscopic views

**Fig. 2:**
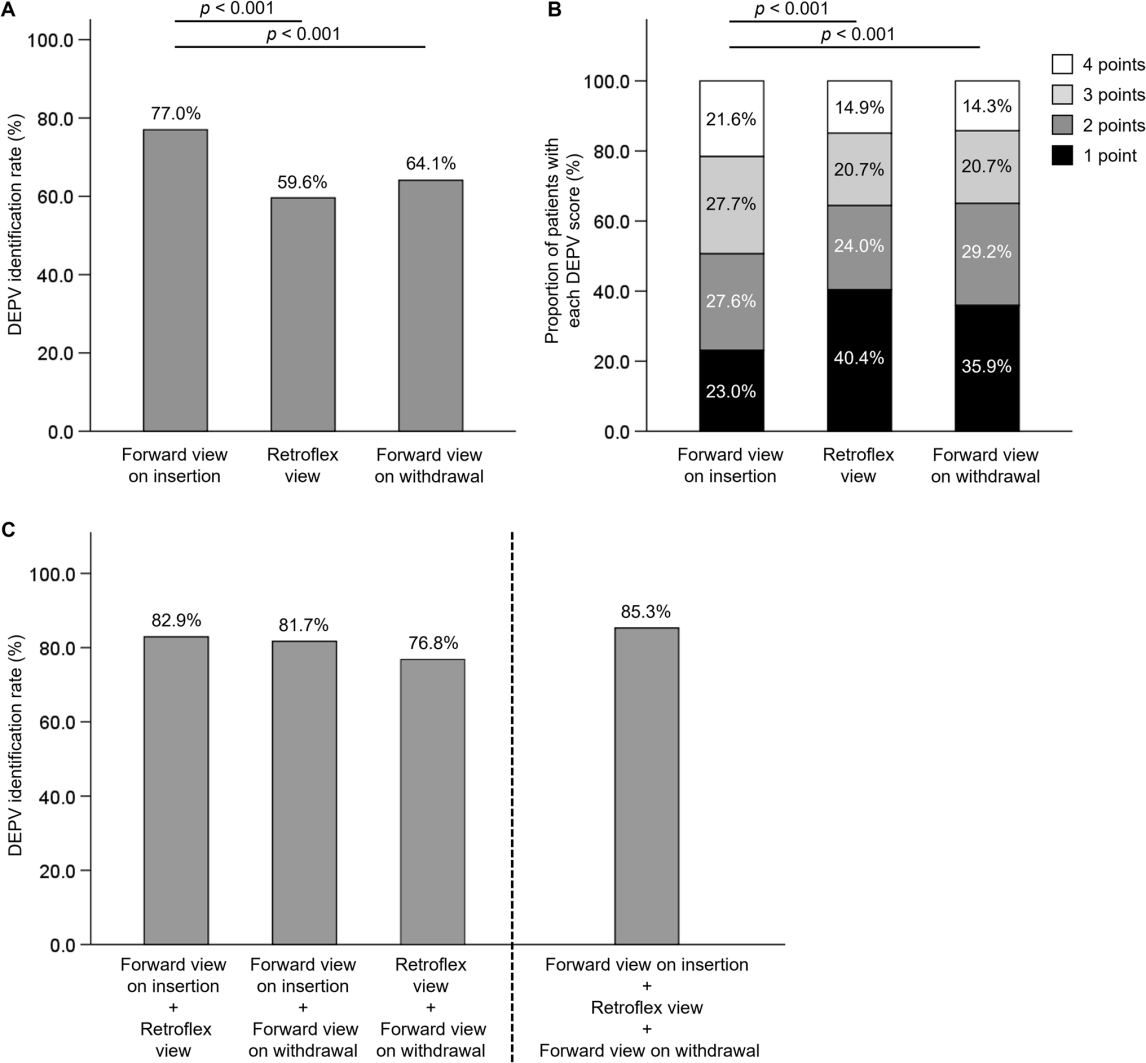
DEPV identification and DEPV score in three endoscopic views. **A** DEPV identification rates. **B** Proportion of patients with each DEPV score. **C** DEPV identification rates when combining two or three views. The DEPV identification rate in forward view on insertion was significantly higher than in the other two views (both, *p* < 0.001). The distribution of the DEPV scores also differed significantly between the forward view on insertion and the other two views (both, *p* < 0.001). The overall DEPV identification rate increased to 85.3% when all three views were combined. DEPV, distal end of palisade vessels

The DEPV identification rate in the forward view on insertion (77.0%) was significantly higher than in the retroflex view (59.6%) and the forward view on withdrawal (64.1%) (both, *p* < 0.001) (Fig. 2A). A similar pattern was observed for a DEPV score of ≥ 3, with higher rates in the forward view on insertion (49.4%) than in the retroflex view (35.6%) and the forward view on withdrawal (35.0%) (both, *p* < 0.001) (Supplementary Fig. S2). The distribution of DEPV scores in the forward view on insertion is shown in Fig. 2B. To explore the difference in identification rates between the forward views on insertion and withdrawal, we compared the rates of complete GEJ distention. Complete GEJ distention was more frequently achieved in the forward view on insertion (73.4% vs. 55.8%; *p* < 0.001) (Supplementary Fig. S3A). Moreover, although high DEPV identification rates were observed in both views when complete distention was present (87.8% vs. 86.8%), the rate was higher in the forward view when limited to cases with incomplete distention (47.1% vs. 35.5%; *p* = 0.017) (Supplementary Fig. S3B and C).

When DEPV identification was unsuccessful in the forward view on insertion, the addition of the retroflex view and forward view on withdrawal increased the DEPV identification in 6.0% and 4.7% of patients, respectively (Supplementary Fig. S4A and S4B). Consequently, using all three views increased the overall identification rate to 85.3% (Fig. 2C) and the proportion of cases with DEPV scores ≥ 3 to 61.8% (Supplementary Fig. S2).

**Fig. 3:**
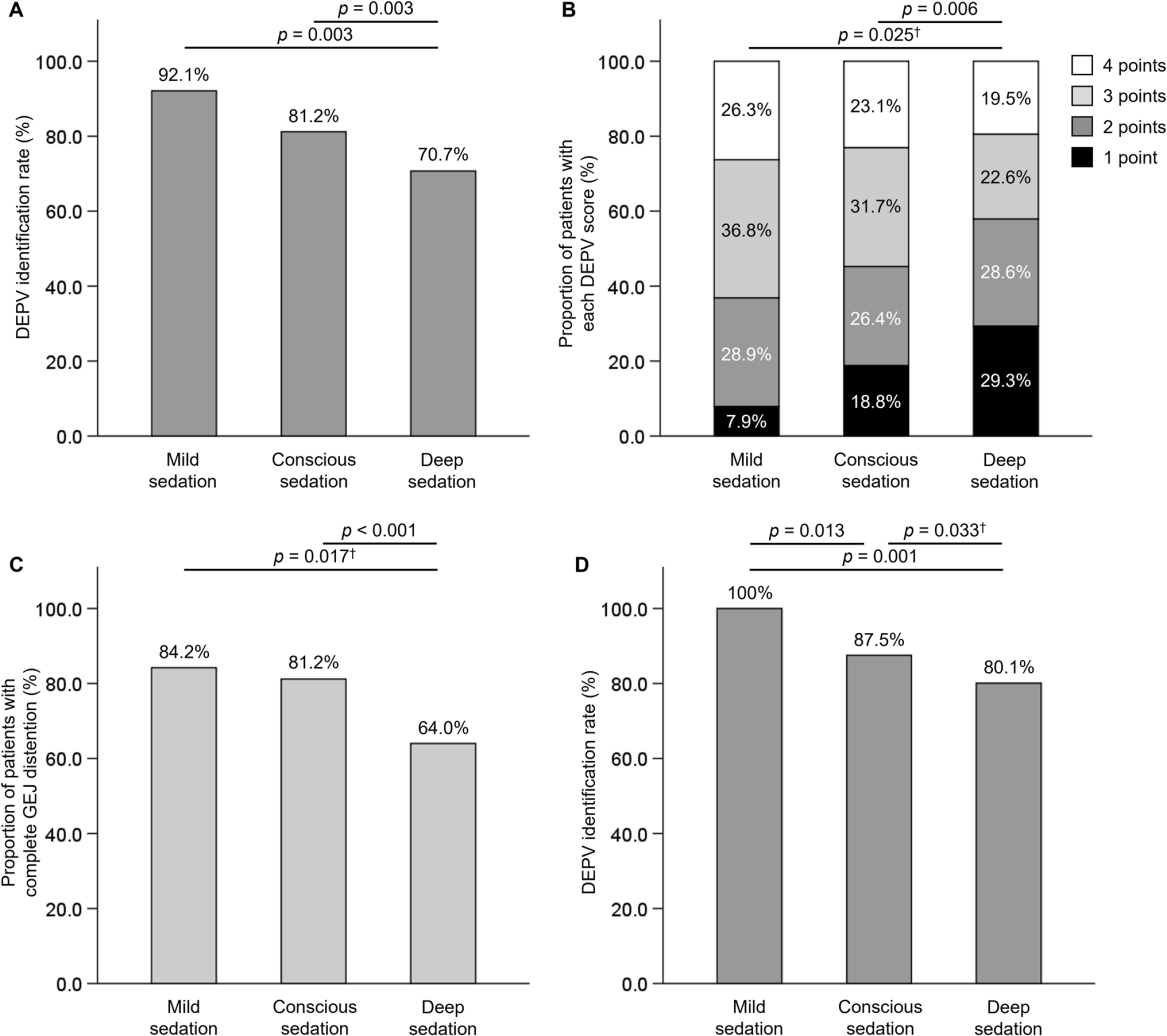
DEPV identification and DEPV scores according to sedation level. **A** DEPV identification rates in the forward view on insertion. **B** Proportion of patients with each DEPV score in the forward view on insertion. **C** Proportion of patients with complete GEJ distention in the forward view on insertion. **D** DEPV identification rates when combining three views. † Statistical significance was removed after Bonferroni correction. DEPV, distal end of palisade vessels

The DEPV identification rates were 92.1%, 81.2%, and 70.7% in minimal, conscious, and deep sedations, respectively (Fig. 3A). The proportions of cases with DEPV scores ≥ 3 were 63.2%, 54.8%, and 42.1% at each sedation level (Supplementary Fig. S5A). The distribution of DEPV scores differed significantly between conscious and deep sedation (*p* = 0.006), although no significant differences were observed between minimal and conscious or deep sedation, due to the small number of patients in the minimal sedation group (Fig. 3B). Complete GEJ distention was more frequently observed under conscious sedation (81.2%) than under deep sedation (64.0%) (*p* < 0.001) (Fig. 3C). When all three views were combined, the DEPV identification rates reached 100%, 87.5%, and 80.1% in minimal, conscious, and deep sedation, respectively (Fig. 3D). The proportions of cases with DEPV scores ≥ 3 increased to 84.2%, 67.0%, and 53.5%, respectively (Supplementary Fig. S5B).

### Factors associated with unsuccessful DEPV identification

In the univariate analysis, portal hypertension, deep sedation, analgesic use, reflux esophagitis LA grade B/C, hiatus hernia, and incomplete GEJ distention were associated with unsuccessful DEPV identification (Table 2). The multivariate analysis revealed deep sedation (odds ratio [OR], 3.83; 95% confidence interval [CI] 1.05–14.02), reflux esophagitis with minimal change (OR, 2.24; 95% CI, 1.18–4.24), LA grade A (OR, 2.28; 95% CI, 1.09–4.76), LA grade B/C (OR, 16.37; 95% CI, 5.42–49.48), and incomplete GEJ distention (OR, 8.87; 95% CI, 5.58–14.10) as risk factors and hiatus hernia (OR, 0.52; 95% CI, 0.28–0.97) as a protective factor. (Table 2). Multicollinearity was not detected in the multivariate model (VIF, 1.04–4.83).

**Table 2:** Factors associated with unsuccessful DEPV identification in forward view on insertion

				Crude		Adjusted	
		No. of patients	No. of events	OR (95% CI)	*p* value	OR (95% CI)	*p* value
Age, years	< 75	448	99	1.00 (Reference)		1.00 (Reference)	
	≥ 75	190	48	1.19 (0.80–1.77)	0.386	1.16 (0.70–1.94)	0.565
Sex	Female	301	59	1.00 (Reference)		1.00 (Reference)	
	Male	337	88	1.45 (0.997–2.11)	0.052	1.20 (0.76–1.90)	0.424
BMI, kg/m^2^	< 18.5	67	10	0.62 (0.31–1.26)	0.187	0.58 (0.25–1.31)	0.188
	≥ 18.5, < 25.0	431	95	1.00 (Reference)		1.00 (Reference)	
	≥ 25.0	140	42	1.52 (0.99–2.32)	0.056	1.49 (0.89–2.51)	0.131
Portal hypertension	No	623	140	1.00 (Reference)		1.00 (Reference)	
	Yes	15	7	3.02 (1.08–8.47)	0.036	3.21 (0.90–11.40)	0.071
PPI/P-CAB use	No	469	114	1.00 (Reference)		1.00 (Reference)	
	Yes	169	33	0.76 (0.49–1.17)	0.207	0.87 (0.52–1.46)	0.603
Sedation level	Minimal	38	3	1.00 (Reference)		1.00 (Reference)	
	Conscious	303	57	2.70 (0.80–9.10)	0.108	2.73 (0.74–10.05)	0.131
	Deep	297	87	4.83 (1.45–16.13)	0.010	3.83 (1.05–14.02)	0.043
Analgesic use	No	414	85	1.00 (Reference)		1.00 (Reference)	
	Yes	224	62	1.48 (1.02–2.16)	0.041	1.02 (0.61–1.72)	0.934
Antispasmodic use	No	580	134	1.00 (Reference)		1.00 (Reference)	
	Yes	58	13	0.96 (0.50–1.84)	0.905	0.67 (0.30–1.52)	0.340
Type of endoscope	Standard	624	144	1.00 (Reference)		1.00 (Reference)	
	Ultrathin	14	3	0.91 (0.25–3.30)	0.895	1.18 (0.29–4.81)	0.816
Reflux esophagitis	None	480	97	1.00 (Reference)		1.00 (Reference)	
	Minimal change	83	22	1.42 (0.83–2.43)	0.196	2.24 (1.18–4.24)	0.013
	LA grade A	57	17	1.68 (0.91–3.09)	0.096	2.28 (1.09–4.76)	0.028
	LA grade B/C	18	11	6.21 (2.34–16.43)	< 0.001	16.37 (5.42–49.48)	< 0.001
Hiatus hernia	No	502	125	1.00 (Reference)		1.00 (Reference)	
	Yes	136	22	0.58 (0.35–0.96)	0.034	0.52 (0.28–0.97)	0.038
Incomplete GEJ distention	No	468	57	1.00 (Reference)		1.00 (Reference)	
	Yes	170	90	8.11 (5.39–12.21)	< 0.001	8.87 (5.58–14.10)	< 0.001
Endoscopic gastric atrophy	No	319	74	1.00 (Reference)		1.00 (Reference)	
	Closed type	131	25	0.78 (0.47–1.30)	0.339	0.62 (0.34–1.14)	0.125
	Open type	188	48	1.14 (0.75–1.73)	0.553	1.03 (0.59–1.79)	0.919

DEPV, distal end of palisade vessels; OR, odds ratio; CI, confidence interval; BMI, body mass index; PPI, proton pump inhibitor; P-CAB, potassium-competitive acid blocker; LA, Los Angeles; GEJ, gastroesophageal junction

### Concordance rate in the PEGV and DEPV and factors associated with discordance

**Fig. 4:**
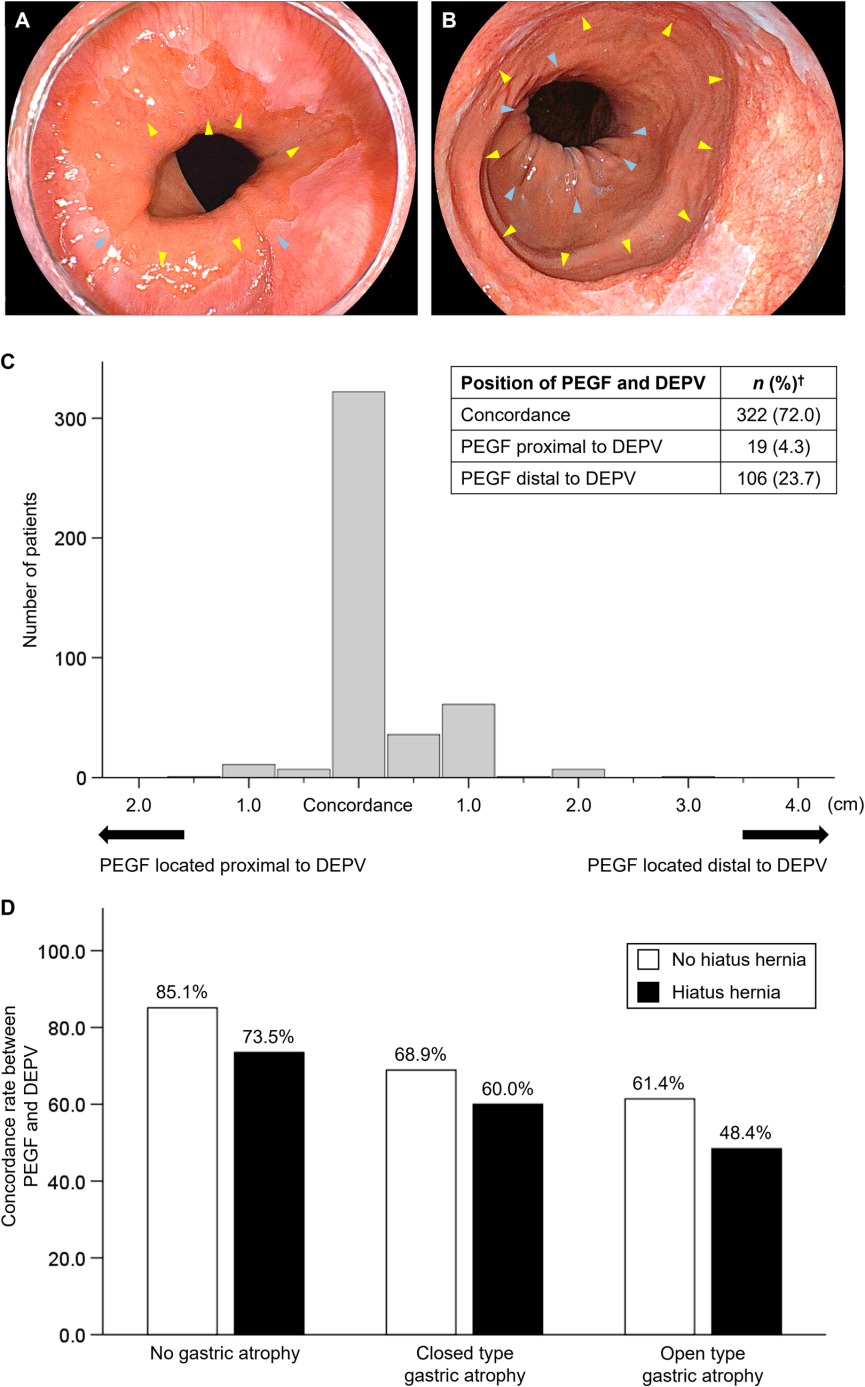
Position of PEGF relative to DEPV. **A** Endoscopic image of a case with PEGF located proximal to DEPV (light blue arrow: PEGF, yellow arrow: DEPV). **B** Endoscopic image of a case with PEGF located distal to DEPV (light blue arrow: PEGF, yellow arrow: DEPV). **C** Position of the PEGF relative to the DEPV. **D** Concordance rate between PEGF and DEPV according to the presence of gastric atrophy and hiatus hernia. † A total of 191 cases were excluded from this analysis due to unsuccessful identification of the DEPV (*n* = 147) followed by that of the PEGF (*n* = 44). The concordance rate between PEGF and DEPV was 72.0%. According to gastric atrophy and hiatus hernia status, the rate ranged from 48.4% to 85.1%. PEGF, proximal end of the gastric folds; DEPV, distal end of palisade vessels

After exclusion of 191 patients due to unsuccessful identification of the DEPV (*n* = 147) followed by that of the PEGF (*n* = 44), 447 patients were analyzed for concordance between the PEGF and DEPV. A total of 322 patients (72.0%) showed concordance, whereas PEGF was located proximal and distal to DEPV in 19 (4.3%) and 106 (23.7%) patients, respectively (Fig. 4A–C).

In the univariate analysis, hiatus hernia and closed- and open-type gastric atrophy were significantly associated with discordance (Table 3). In the multivariate analysis, independent risk factors for discordance were use of ultrathin endoscope (OR, 6.20; 95% CI, 1.61–23.92), hiatus hernia (OR, 1.87; 95% CI, 1.10–3.15), closed-type gastric atrophy (OR, 2.50; 95% CI, 1.40–4.46), and open-type gastric atrophy (OR, 3.49; 95% CI, 1.96–6.21) (Table 3). No evidence of multicollinearity was observed in the multivariate model (VIF, 1.03–4.06).

**Table 3:** Factors associated with discordance of PEGF and DEPV in forward view on insertion

				Crude		Adjusted	
		No. of patients	No. of events	OR (95% CI)	*p* value	OR (95% CI)	*p* value
Age, years	< 75	323	85	1.00 (Reference)		1.00 (Reference)	
	≥ 75	124	40	1.33 (0.85–2.09)	0.211	0.80 (0.47–1.36)	0.405
Sex	Female	229	61	1.00 (Reference)		1.00 (Reference)	
	Male	218	64	1.15 (0.76–1.73)	0.522	0.98 (0.62–1.54)	0.917
BMI, kg/m^2^	< 18.5	51	13	0.86 (0.44–1.68)	0.651	0.75 (0.36–1.57)	0.443
	≥ 18.5, < 25.0	308	88	1.00 (Reference)		1.00 (Reference)	
	≥ 25.0	88	24	0.94 (0.55–1.59)	0.811	0.79 (0.44–1.40)	0.415
Portal hypertension	No	439	123	1.00 (Reference)		1.00 (Reference)	
	Yes	8	2	0.86 (0.17–4.30)	0.851	1.09 (0.20–5.86)	0.922
PPI/P-CAB use	No	320	90	1.00 (Reference)		1.00 (Reference)	
	Yes	127	35	0.97 (0.61–1.54)	0.904	0.83 (0.50–1.37)	0.456
Sedation level	Minimal	34	7	1.00 (Reference)		1.00 (Reference)	
	Conscious	231	60	1.35 (0.56–3.27)	0.501	1.53 (0.61–3.86)	0.369
	Deep	182	58	1.80 (0.74–4.38)	0.193	1.58 (0.61–4.09)	0.344
Analgesic use	No	310	80	1.00 (Reference)		1.00 (Reference)	
	Yes	137	45	1.41 (0.91–2.18)	0.127	1.34 (0.77–2.32)	0.301
Antispasmodic use	No	409	110	1.00 (Reference)		1.00 (Reference)	
	Yes	38	15	1.77 (0.89–3.52)	0.102	1.13 (0.49–2.60)	0.770
Type of endoscope	Standard	437	120	1.00 (Reference)		1.00 (Reference)	
	Ultrathin	10	5	2.64 (0.75–9.29)	0.130	6.20 (1.61–23.92)	0.008
Reflux esophagitis	None	348	100	1.00 (Reference)		1.00 (Reference)	
	Minimal change	55	12	0.69 (0.35–1.37)	0.289	0.67 (0.33–1.38)	0.277
	LA grade A	37	9	0.80 (0.36–1.75)	0.572	0.74 (0.32–1.74)	0.494
	LA grade B/C	7	4	3.31 (0.73–15.04)	0.122	2.44 (0.49–12.23)	0.279
Hiatus hernia	No	346	87	1.00 (Reference)		1.00 (Reference)	
	Yes	101	38	1.80 (1.12–2.87)	0.015	1.87 (1.10–3.15)	0.020
Incomplete GEJ distention	No	377	110	1.00 (Reference)		1.00 (Reference)	
	Yes	70	15	0.66 (0.36–1.22)	0.187	0.58 (0.30–1.13)	0.109
Endoscopic gastric atrophy	No	233	44	1.00 (Reference)		1.00 (Reference)	
	Closed type	94	31	2.11 (1.23–3.63)	0.007	2.50 (1.40–4.46)	0.002
	Open type	120	50	3.07 (1.88–5.00)	< 0.001	3.49 (1.96–6.21)	< 0.001

PEGF, proximal end of the gastric folds; DEPV, distal end of palisade vessels; OR, odds ratio; CI, confidence interval; BMI, body mass index; PPI, proton pump inhibitor; P-CAB, potassium-competitive acid blocker; LA, Los Angeles; GEJ, gastroesophageal junction

Because an ultrathin endoscope was used in only a small number of patients, we further analyzed concordance rates between DEPV and PEGF according to the presence of gastric atrophy and hiatus hernia in patients examined with a standard endoscope. The concordance rate was 85.1% in patients without hiatus hernia and gastric atrophy, but decreased to 48.4% in those with both hiatus hernia and open type gastric atrophy (Fig. 4D).

## Discussion

This large-scale multicenter prospective study demonstrated that the DEPV identification rate was highest in the forward view on insertion (77.0%) among the three endoscopic views. Risk factors for unsuccessful DEPV identification included deep sedation, reflux esophagitis, and incomplete GEJ distention, whereas hiatal hernia was identified as a protective factor. In addition, concordance between PEGF and DEPV was 72.0%, and discordance was significantly associated with gastric atrophy, hiatus hernia, and the use of ultrathin endoscopes.

The forward view on insertion significantly outperformed retroflex and withdrawal views for identifying DEPV. The difference between the forward view on insertion and withdrawal is partly attributable to the disparity in complete GEJ distention (73.4% vs. 55.8%), which was associated with unsuccessful DEPV identification. Although the underlying reason remains unclear, mechanical irritation from the endoscope during EGD observation might have contributed to incomplete GEJ distention on withdrawal. Furthermore, the combined use of all three views increased the overall identification rate to 85.3%, although the proportion of cases with DEPV scores ≥ 3 was 61.8%. These findings indicate that DEPV can be identified, at least partially, under real-world conditions and support prioritizing the forward view on insertion as the standard method, with additional views serving as complementary strategies when the DEPV was not identified in the forward view on insertion. This evidence refines previous consensus statements [7], which regarded both forward and retroflex views as appropriate for DEPV identification, and may help standardize the endoscopic assessment of the GEJ.

Sedation level was a critical determinant of DEPV visibility. Deep sedation emerged as an independent risk factor for unsuccessful identification, whereas minimal sedation was associated with markedly higher visualization rates. Notably, minimal sedation enabled 100% DEPV identification and DEPV scores ≥ 3 in 84.2% of cases when all three views were combined. This may be partially explained by the fact that deeper sedation reduces patient compliance with instructions such as deep inspiration, which enhances visualization of PV and also impairs adequate GEJ distention. Although this study did not directly evaluate compliance with such instructions, an inability to take deep inspiration, rather than deeper sedation itself, may be the true risk factor for unsuccessful DEPV identification. Furthermore, reflux esophagitis and incomplete GEJ distention were identified as risk factors, reflecting the difficulty of detecting thin longitudinal vessels in inflamed or poorly distended mucosa [14]. In contrast, hiatus hernia facilitated DEPV identification, probably by improving mucosal distention and making the vascular structures more visible. These findings highlight the importance of considering sedation depth and associated conditions when planning or interpreting GEJ examination.

Concordance between PEGF and DEPV was moderate (72.0%), and PEGF was distal to DEPV in nearly one-quarter of patients. Discordance was significantly associated with gastric atrophy and hiatus hernia, in addition to use of ultrathin endoscopes. Although the reason for the association between ultrathin endoscope use and discordance remains unclear, the lower image resolution of ultrathin endoscopes may have contributed to discordance between PEGF and DEPV. Furthermore, gastric atrophy and hiatus hernia likely alter or obscure the gastric folds, reducing their reliability as a landmark. In severe gastric atrophy, gastric folds can become indistinct [7], whereas even mild to moderate atrophic changes, including closed type gastric atrophy, might alter the morphology or apparent position of the gastric folds. In hiatus hernia, cranial displacement of the stomach might further modify the morphology and positional stability of the gastric folds. In contrast, DEPV represents an anatomically fixed landmark corresponding to the GEJ. Thus, PEGF may not be relied on as the sole marker of GEJ, particularly in patients with gastric atrophy or hiatus hernia.

This study has several strengths. It represents the largest multicenter prospective evaluation of DEPV to date, including more than 600 patients from 15 institutions, which allowed a detailed analysis including risk factors for unsuccessful DEPV identification or discordance between PEGF and DEPV. The introduction of a novel DEPV scoring system enabled a semi-quantitative assessment of visibility and systematic evaluation across three observation views. These strengths would provide rigorous evidence for clinical practice.

Nevertheless, several limitations should be acknowledged. First, pathological confirmation of GEJ was not available, as biopsy-based localization remains technically challenging particularly in cases with gastric atrophy [7, 26]. Second, although this was a prospective study, it did not have a randomized design. A randomized controlled study is required to more robustly evaluate the effects of observation views and sedation levels on DEPV identification. Third, although our study demonstrated the effect of deeper sedation on DEPV identification, we did not compare identification rates under unsedated conditions. A multicenter prospective study addressing this issue is currently ongoing (UMIN000052827). Fourth, since there are no established definitions for GEJ distention, its assessment was subjective in this study. Fifth, this study did not comprehensively evaluate PEGF identification rates, preventing a direct head-to-head comparison of absolute performance between the two landmarks. Sixth, examiner variability, despite prior training before starting this study, cannot be fully excluded. Furthermore, as most examiners were expert endoscopists, the applicability of these results to non-expert endoscopists remains uncertain. In addition, their generalizability to Western endoscopists is unclear. Finally, events per variable in the multivariate analyses were < 10, which may have limited the statistical power of the analyses [27].

In conclusion, this large-scale multicenter prospective study established a standardized method for DEPV observation under sedation, prioritizing the forward view on insertion, with retroflex and withdrawal views as needed, yielding an overall identification rate of 85.3%. Given that deep sedation was an independent risk factor for unsuccessful DEPV identification, deep sedation should be avoided when accurate GEJ landmarking is required, and DEPV should be identified early during the insertion view, which was the most effective for visualization. In addition, concordance between PEGF and DEPV was incomplete and influenced by gastric atrophy and hiatus hernia, highlighting the limitations of PEGF. These findings support the feasibility of DEPV as a practical landmark for routine GEJ assessment.

## Supplementary Information

Below is the link to the electronic supplementary material.

Supplementary file1 (DOCX 486 KB)

## Abbreviations

BMI: Body mass index
CI: Confidence interval
DEPV: Distal end of palisade vessels
GEJ: Gastroesophageal junction
IQR: Interquartile range
LA: Los Angeles
EGD: Esophagogastroduodenoscopy
OR: Odds ratio
P-CAB: Potassium-competitive acid blocker
PEGF: Proximal end of gastric folds
PPI: Proton pump inhibitor
PV: Palisade vessels
STROBE: Strengthening the Reporting of Observational Studies in Epidemiology
VIF: Variance inflation factors
