# Supplementary material for: Identification of the distal end of the palisade vessels under sedation: a multicenter prospective study in Japan
Source: J Gastroenterol. 2026 Apr 1;61(6):719–31. doi: 10.1007/s00535-026-02385-6 (PMC13219092; doi:10.1007/s00535-026-02385-6)
Supplement: Supplementary file 1 — Supplementary file1 (DOCX 486 KB) [file 535_2026_2385_MOESM1_ESM.docx]

**
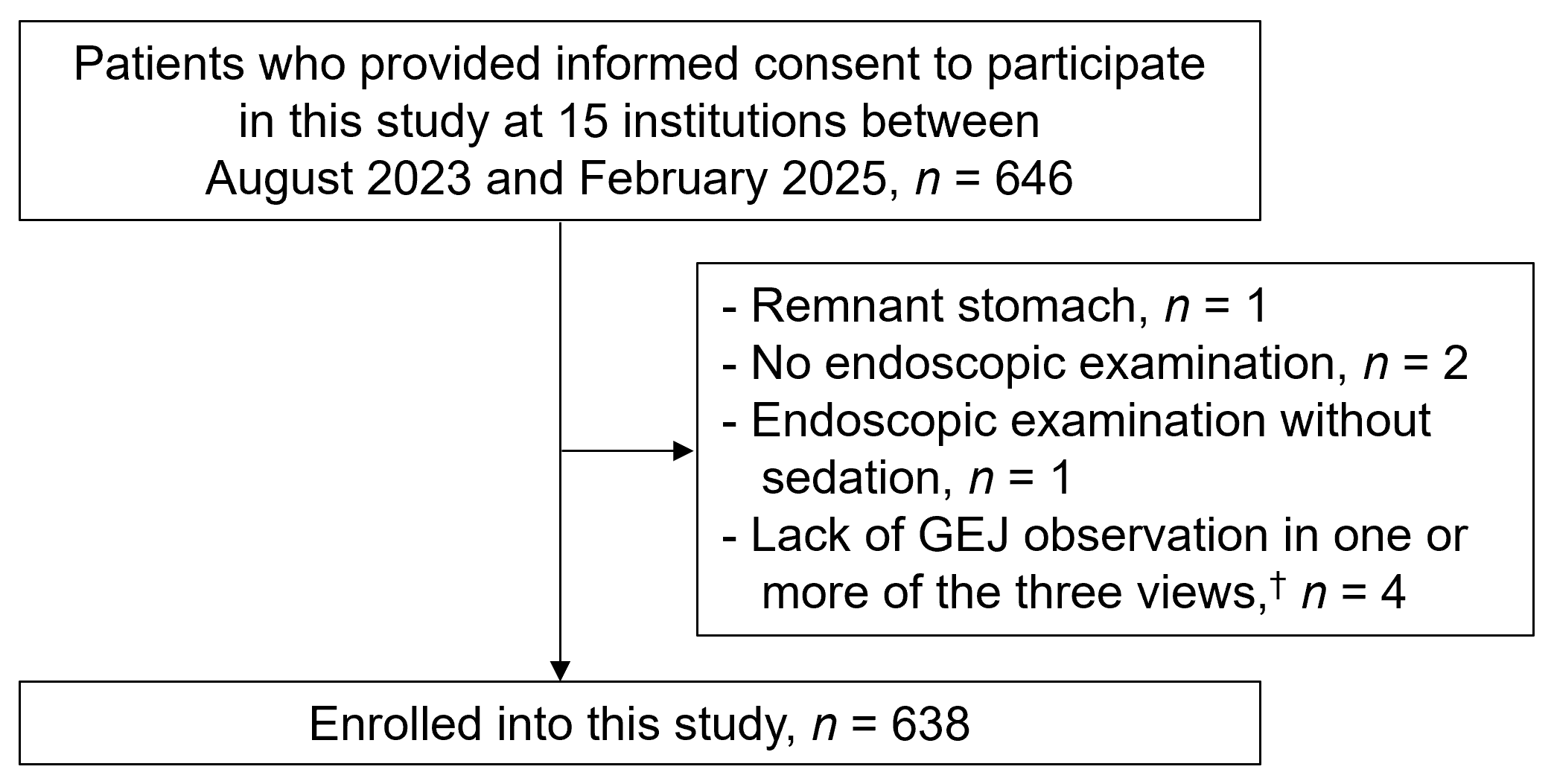
**

**Supplementary Fig. 1.** Flow diagram of the enrolled patients

† Forward view on insertion, retroflex view, and forward view on withdrawal.

GEJ, gastroesophageal junction.

**
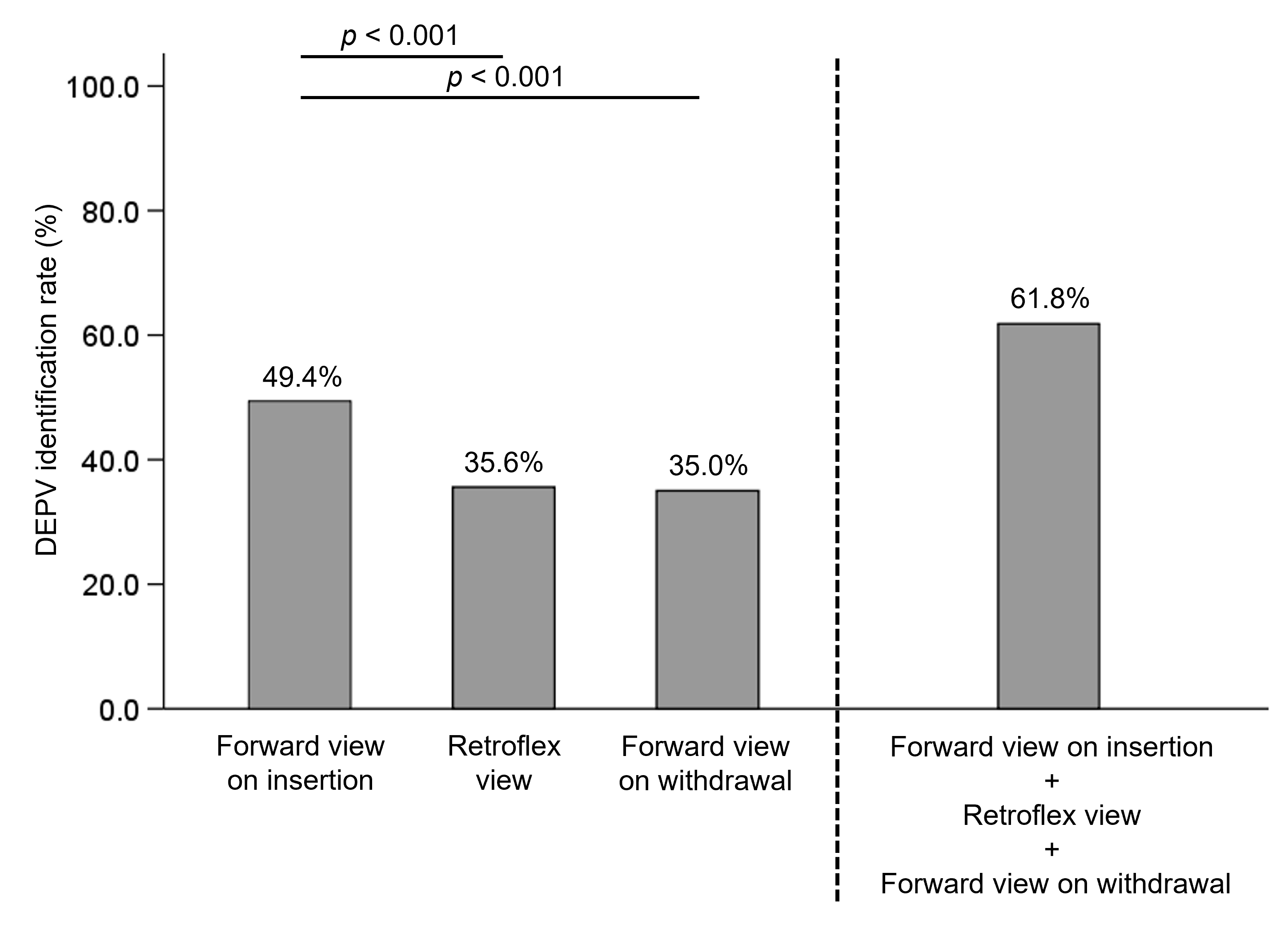
**

**Supplementary Fig. 2.** Rates of DEPV scores ≥ 3 in three endoscopic views

The rate of DEPV scores ≥ 3 in forward view on insertion was significantly higher than in the other two views (both, *p* < 0.001). The overall rate of DEPV scores ≥ 3 increased to 61.8% when all three views were combined.

DEPV, distal end of palisade vessels**.**

**
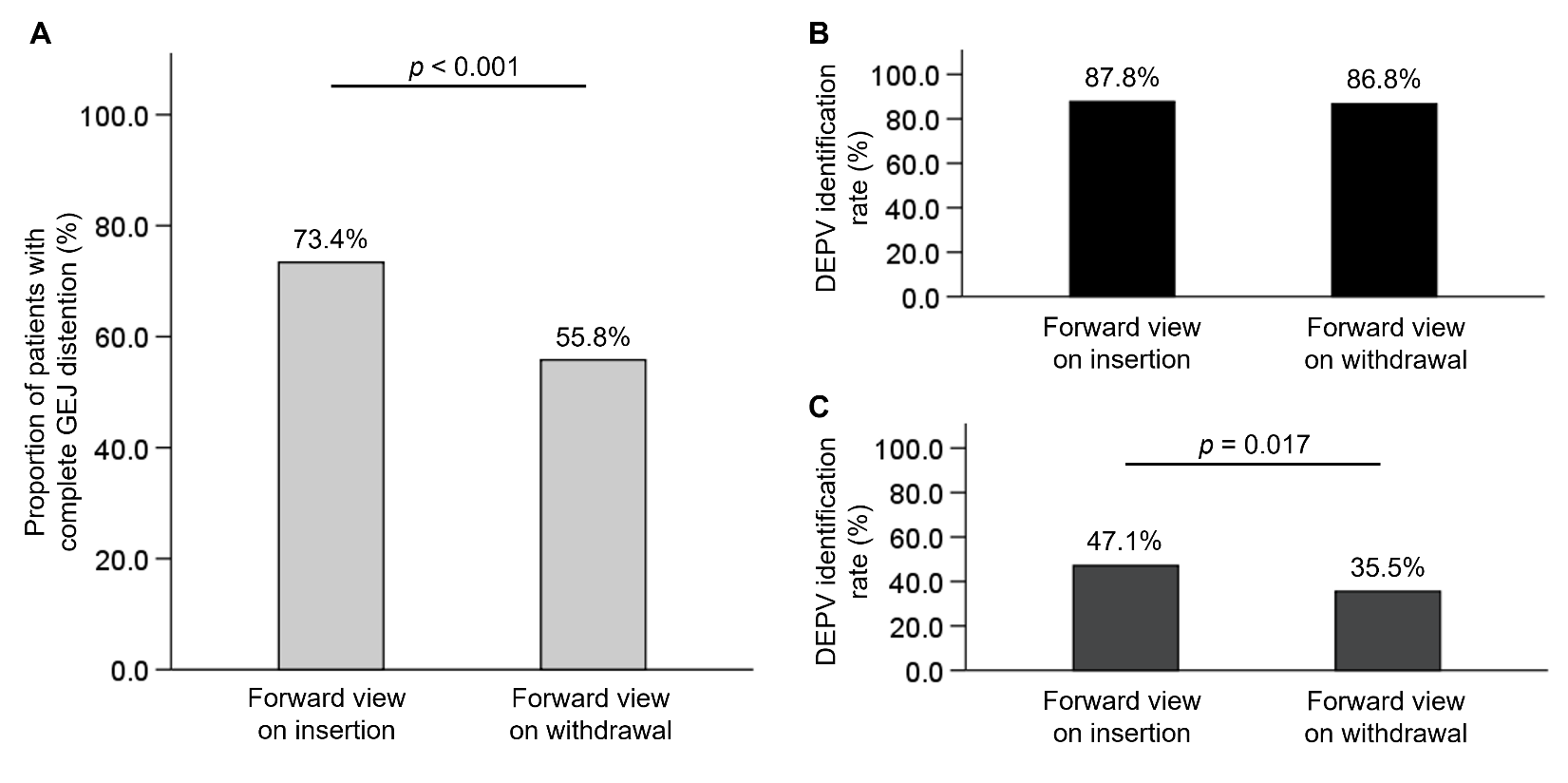
Supplementary Fig. 3.** Effect of GEJ distention on DEPV identification in the forward view using a standard endoscope

1. Proportion of patients with complete GEJ distention
2. DEPV identification rate in patients with complete GEJ distention
3. DEPV identification rate in patients with incomplete GEJ distention

Complete GEJ distention was more frequently achieved in the forward view on insertion than in the forward view on withdrawal (*p* < 0.001). DEPV identification rates were high in both views when GEJ distention was complete; however, when distention was incomplete, the rate was significantly lower in the forward view on withdrawal than in the forward view on insertion (*p* < 0.05).

GEJ, gastroesophageal junction; DEPV, distal end of palisade vessels.

**
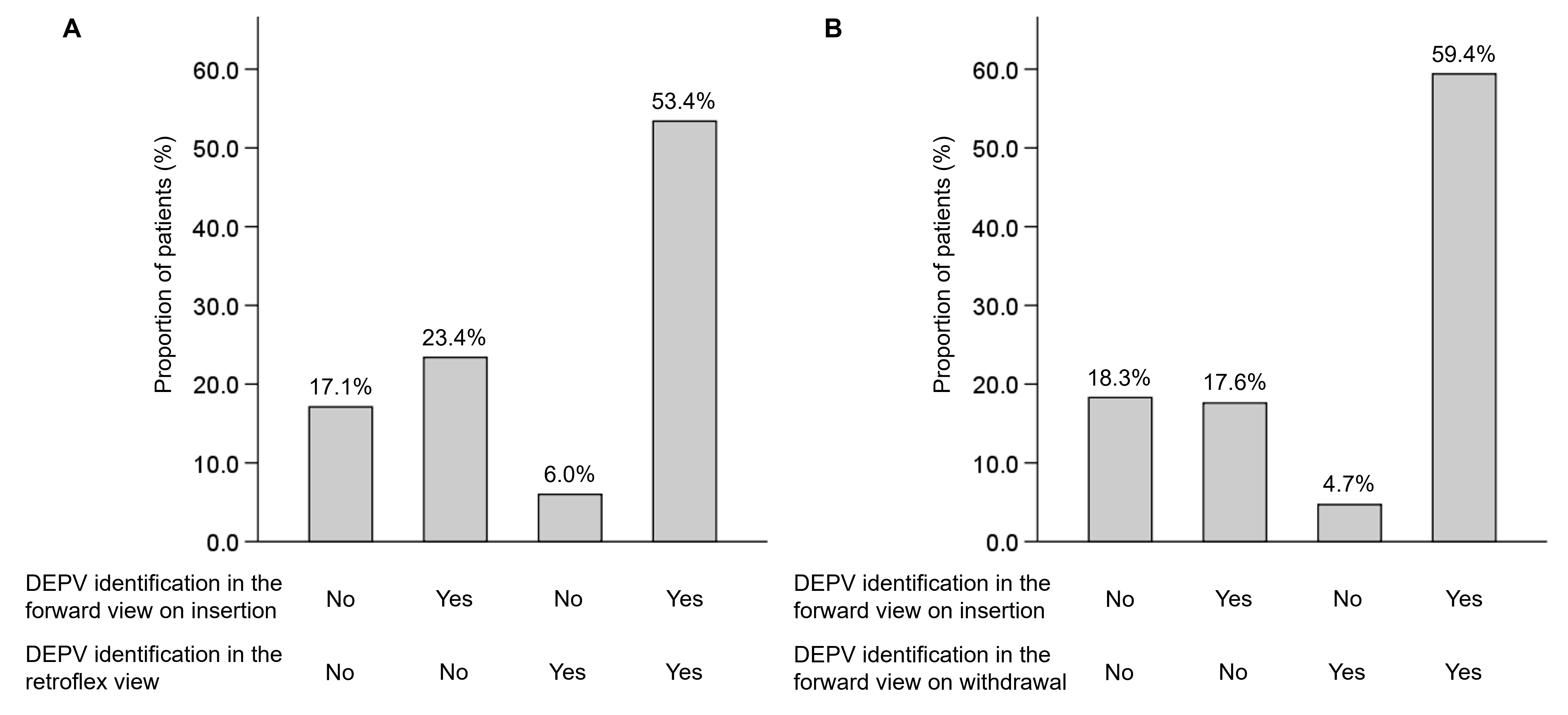
Supplementary Fig. 4.** Distribution of patients according to DEPV identification across the three views

1. Forward view on insertion vs. retroflex view
2. Forward view on insertion vs. forward view on withdrawal

DEPV, distal end of palisade vessels.

**
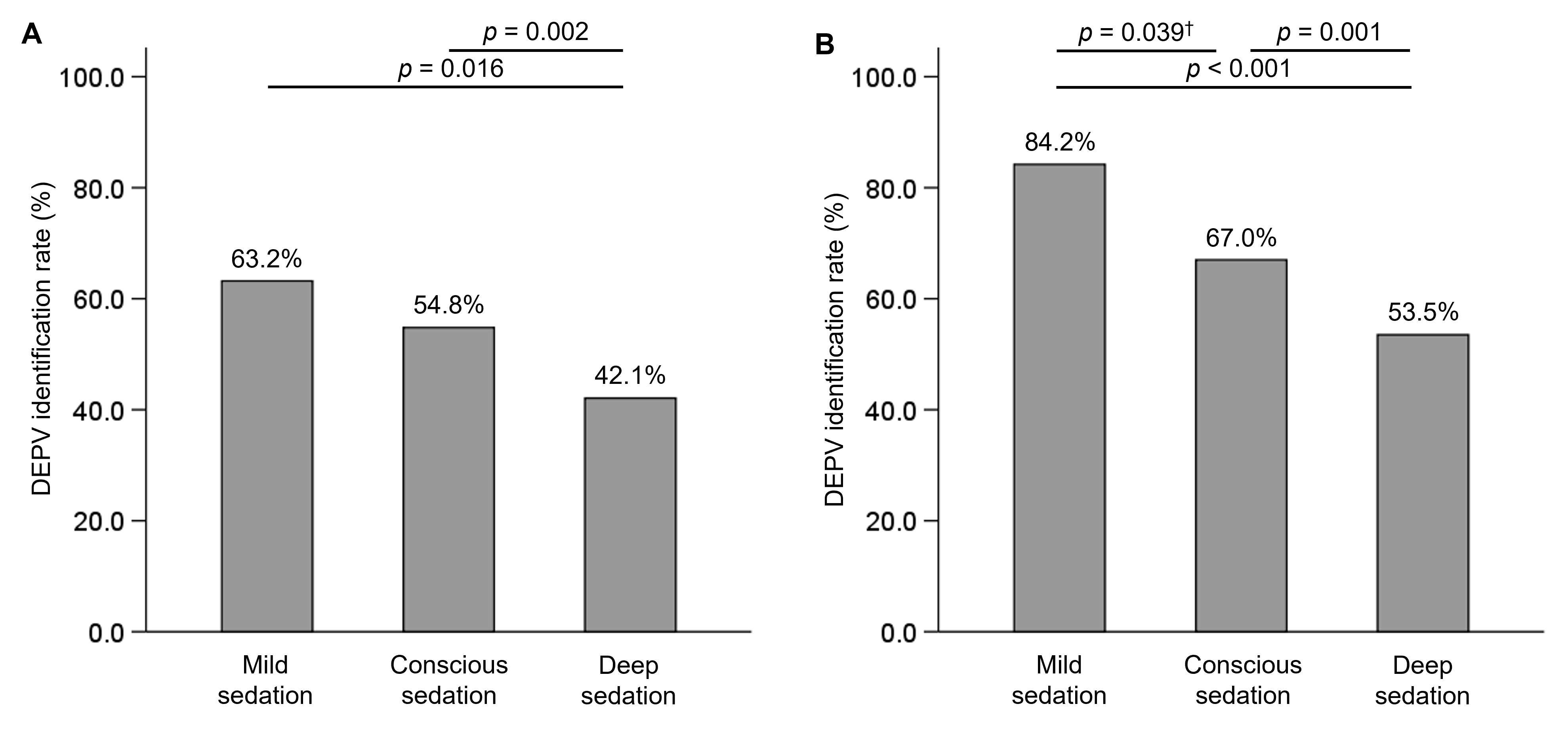
Supplementary Fig. 5.** Rates of DEPV scores ≥ 3 according to sedation level

1. Rates of DEPV scores ≥ 3 in forward view on insertion
2. Rates of DEPV scores ≥ 3 when combining three views

† Statistical significance was removed after Bonferroni correction.

DEPV, distal end of palisade vessels.
